# Pulling chromatin apart: Unstacking or Unwrapping?

**DOI:** 10.1186/2046-1682-5-21

**Published:** 2012-11-27

**Authors:** Jean Marc Victor, Jordanka Zlatanova, Maria Barbi, Julien Mozziconacci

**Affiliations:** 1, Laboratory for Theoretical Physics of Condensed Matter, UPMC, 75005 Paris, France; 2 Department of Molecular Biology, University of Wyoming, Laramie, WY 82071, USA

## Abstract

**Background:**

Understanding the mechanical properties of chromatin is an essential step towards deciphering the physical rules of gene regulation. In the past ten years, many single molecule experiments have been carried out, and high resolution measurements of the chromatin fiber stiffness are now available. Simulations have been used in order to link those measurements with structural cues, but so far no clear agreement among different groups has been reached.

**Results:**

We revisit here some of the most precise experimental results obtained with carefully reconstituted fibers.

**Conclusions:**

We show that the mechanical properties of the chromatin fiber can be quantitatively accounted for by the stiffness of the DNA molecule and the 3D structure of the chromatin fiber.

## Background

In the nucleus of eukaryotic cells, chromatin is constantly under mechanical stresses due to DNA transcription and replication. In order to better understand how nucleosomal arrays can deal with such stress, several groups have used optical and magnetic tweezers to probe the response of native or reconstituted arrays to stretch and torque. In most of these studies, the only experimental read out is the extension of the array, and modelling efforts are often needed to interpret these results in term of structures. Ten years of work have revealed essential features of the mechanical response of the chromatin fiber to external stress (for two recent reviews see [1,2]). In one of the most recent, and certainly one of the most accurate study, Kruithof and colleagues used magnetic tweezers to probe the mechanical properties of reconstituted chromatin fibers under physiological ionic conditions at an unprecedented resolution [3]. Their study revealed that a regular nucleosome array containing linker histones (LH) is a very soft structure (a soft spring) which can be easily stretched up to three times its resting length. They interpreted these results to support a model of the 30 nm fiber in which nucleosomes are stacked in a helical structure reminiscent of the solenoid model proposed by Finch and Klug decades ago [4]. Theses new results were reinterpreted in two consecutive modelling studies, one of which agrees with their interpretation [5] whereas the other one does not [6]. Here, we propose an alternative explanation for the Kruithof *et al.* results which is in very good quantitative agreement with their measurements. In a first part of this paper, we will interpret their results in the geometrical framework of the zig-zag model instead of the solenoidal model of chromatin. In a second part, we will see that this alternative explanation can quantitatively account for the fiber stiffness they measure.

## Results and discussion

Kruithof *et al.* assume that the nucleosomes in the chromatin fiber are stacked in a one-start helix. This assumption is mainly based on the spring-like behaviour of the array until it reaches an extension of 150 nm (i.e., three times the resting length of their reconstituted fiber): they reason that this extension may correspond to a fully stretched column of nucleosomes stacked upon each other. When this column is further stretched, the response is characteristic of a disruption of some contacts stabilizing the structure; this disruption is interpreted as an unstacking of the nucleosomes. In the following discussion, we will argue that the assumption of nucleosome stacking is in contradiction with some of their findings.

First, in the most compact form of the nucleosomal array (with LH and Mg^2+^) they observe a compaction of 5 nucleosomes(nuc)/10 nm (that is 50 nm for 25 nucleosomes) compared to the actually measured compaction of 10 nuc/10 nm based on electron microscopy for the very same construct [7]. They propose that this difference in compaction is due to the formation of an alternate structure in which the helix gyres are not interdigitated as proposed earlier for the 10 nuc/10 nm structure seen in electron microscopy [7]. In this respect, the structure they propose closely matches the solenoid structure in which consecutive nucleosomes in the array are stacked on top of each other [4]. In order to achieve this stacking, the 50 bp DNA linkers joining consecutive nucleosomes have to be dramatically bent. Such bending is usually expected to be achieved through the binding of LHs (in this case H1 or H5) onto the DNA linker. Unexpectedly, Kruithof and colleagues observe a very similar mechanical behaviour of nucleosomal arrays with and without LH when the pulling force is low. They conclude that the compact structure, with bent DNA linkers, can form in the absence of LHs and attribute this possibility to the huge nucleosome stacking interaction energy they estimate from the force–extension curves. Whether or not the stacking energy can override constraints of the persistence length of naked DNA remains to be seen.

Second, in order to stack nucleosomes in a helix that has both the compaction and the stiffness they measure, Kruithof and colleagues propose that the nucleosomes do not interact through their faces, as previously and repeatedly reported [8], but through their flexible tails. At the same time the author assume a conventional face-to-face stacking of the nucleosomes when the fiber is fully stretched into a column, in order to be able to match the size of the column. If the nature of stacking would change when pulling on the array (which seems unrealistic), one would not expect the linear force-extension dependence that has been reported. Of note, the linear force-extension dependence was the main reason for assuming nucleosome stacking in the first place [3].

### Modelling the fiber extension using the two-angle chromatin fiber model

**Figure 1:**
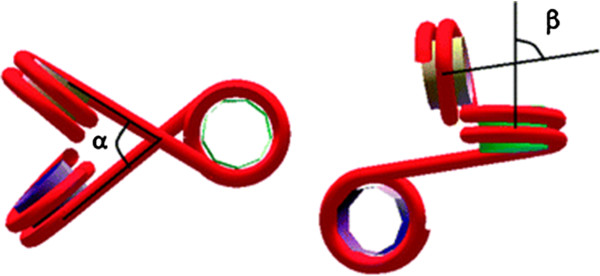
**The two angle model. A** and **B** are two different views of three consecutive nucleosomes in the array. One of the two angles (***α***) is formed between entering and exiting DNA linkers and depends (i) on the presence of LHs and Mg^2+^ ions, and (ii) on the pulling force; the other angle (***β***) determines the relative orientation of one nucleosome to the next and is fixed by the nucleosomal repeat length (NRL).

**Figure 2:**
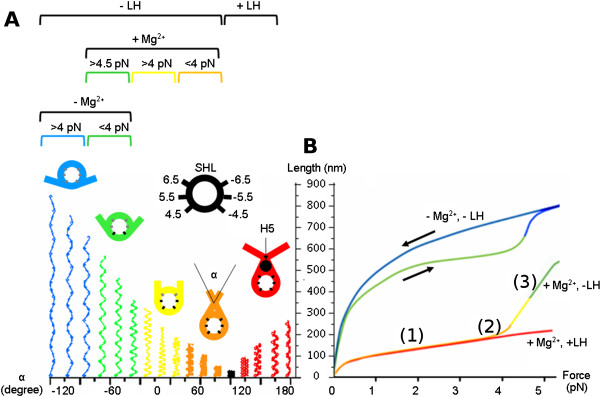
**Model for the array stretching under pulling force. **(**A**) Geometrical model of the array predicted from the two angle model for a NRL of 197 bp for different values of the *α*angle. For each of the five colors (blue, green, yellow, orange and red), the contacts between DNA and the histone core are different. A schematic representation of the location of those contacts (SHL) is presented in black. On each colored nucleosome, SHL are shown in black when there is a physical contact between DNA and the histones and in grey if this contact is disrupted. In blue, the interaction between DNA and the core histones is disrupted at SHL 6.5, 5.5 and 4.5 (resp. -6.5, -5.5 and -4.5). In green, the interaction between DNA and the core histones is disrupted at SHL 6.5 and 5.5 (respectively, -6.5 and -5.5). In yellow, the interaction between DNA and the core histones is disrupted at SHL 6.5 only (respectively, -6.5 ). In orange, no interaction in between DNA and the histones are disrupted. In the red conformation, the linker histone (represented as a black dot) interacts with the entry and exit DNA linker so that the negative crossing is stabilized. (**B**) Summary of the experimental results presented in Kruithof *et al.*[3]. The extension of the array is plotted as a function of the pulling force. The red curve corresponds to the H5 containing array. The orange/yellow/green curve corresponds to the array without LH, in the presence of Mg^2+^. The green/blue curves show the hysteretic behaviour obtained without LH when magnesium has been depleted. The black arrows indicate the curve obtained while increasing (respectively, decreasing) the applied force.

Here, we suggest that the topology and the mechanics of the nucleosomal array can be fully accounted by the well known two-angle model that describes the fiber as an irregular zig-zag structure [9,10] (Figure 1). One of the two angles, *β*, determines the relative orientation of one nucleosome to the next and is roughly fixed by the nucleosomal repeat length (NRL); the other angle, *α*, is formed between entering and exiting DNA linkers and depends in this case (i) on the presence of LHs and Mg^2+^ ions and (ii) on the pulling force. The stretching force can change *α*either through linker bending or the rupture of the contacts between DNA and the histones at the Super-Helical Locations (SHL). We have modelled the array reconstituted on the 197 bp NRL template used in Kruithof *et al.* as a function of the *α*angle (Figure 1A) and compared the array extension with the experimental results of Kruithof *et al.* (Figure 1B). The comparison clearly shows that the experimental observations can be explained in a straightforward way as follows:

(i) In the presence of LHs and Mg^2+^, the entry and exit linkers are crossing and *α*can vary from 120^*o*^to 180^*o*^(in red in Figure 2). This variation allows extension upon stretching from 100 to 250 nm, as observed experimentally.

(ii) In the absence of LHs and in the presence of Mg^2+^, the angle *α*(and hence the array extension) will also depend on the applied force. At forces below 4 pN, *α* can vary from 60^*o*^ (corresponding to the crystal structure of the nucleosome) to 0^*o*^with possible rupture of the weak contacts between DNA and the histones at the SHL 6.5 and -6.5 (in yellow and orange in Figure 2). At higher force (> 4 pN), the strong DNA/histone contacts at SHL 5.5 and -5.5 are progressively disrupted and *α* can decrease from 0^*o*^ to -90^*o*^, leading to a dramatic extension of the array (in yellow and green in Figure 2).

(iii) In the absence of both LHs and Mg^2+^, i.e., when the electrostatic repulsion between the DNA linkers at the entry/exit site is high, *α* is already widely open even at low forces (green in Figure 2). In this case, the contour length of the fiber is longer than 500 nm, and, upon stretching, the array can be smoothly extended up to this length following a worm-like chain behaviour. Upon further force increase, the DNA/histone contacts at SHL -4.5 and 4.5 are eventually disrupted, resulting in further increase of the length up to 700 nm. Kruithof and colleagues propose that this extension is due to nucleosome unstacking. However, similar changes in extension were reported in the case of single nucleosomes and interpreted in terms of unwrapping of DNA from the octamer by the same authors [11,12]. Other labs have also observed DNA unwrapping under low force conditions, both in the fiber context [13] and on individual nucleosomes [14]. We believe that partial unwrapping of DNA in individual nucleosomes in the array is responsible for the observed stretching behaviour of the fiber.

### The stiffness of the nucleosomal array can be explained by the DNA mechanical properties

**Figure 3:**
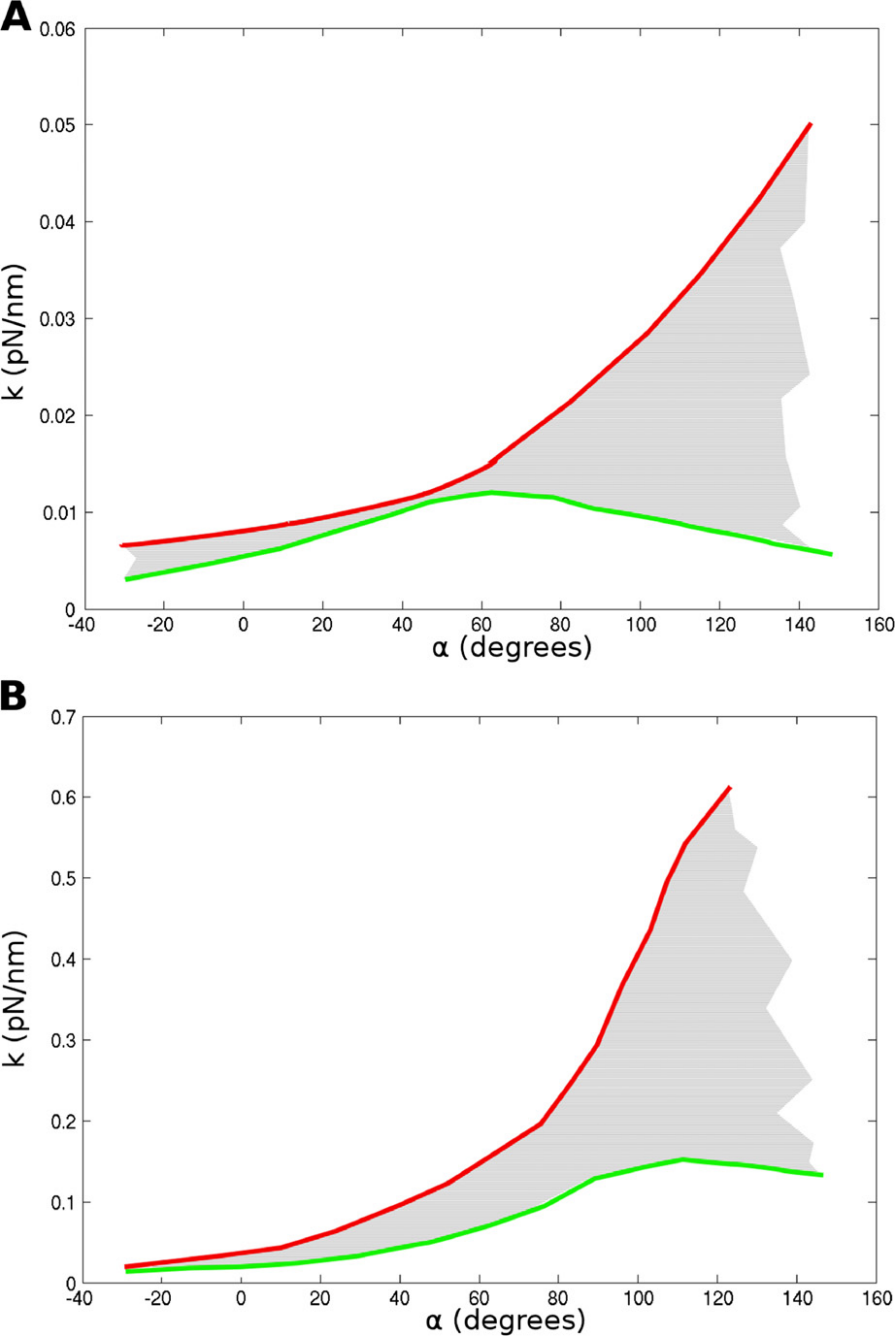
**Values of the array stiffness for different values of the angle (*α*).** In red respectively, green we show the upper respectively, lower value of *k* for different *α*angles. The grey area in between the two curves therefore represents the possible values for the stiffness *k*. Results are shown both for (**A**) the 197 bp NRL and (**B**) the 167 bp NRL. Note that the scales of the Y-axis are different in (**A**) and (**B**).

Importantly, our interpretation of the stretching data is in very good quantitative agreement with other measurements presented in the Kruithof *et al.* paper. The stiffness of the array in the linear extension regime can be estimated as a function of *α*according to our former tunable spring elasticity model [15] (see Methods). For the 197 bp NRL array with Mg^2+^, we found that the stiffness *k* for *α*= 70^*o*^ is close to 0.015 pN/nm (see Figure 3A). This value compares well with the experimental measure, 0.02 pN/nm, given in Table 1 of the paper by Kruithof *et al.*[3]. As measured by Kruithof *et al.* the stiffness is constant in the first regime ((1) on Figure 2B) of the array stretching. This observation is in agreement with the fact that *k* around this value of *α* is roughly constant. In the case where LHs are absent, when the force is further increased, the array becomes more extended and *α* decreases towards zero according to our geometrical model. As can be seen from our calculations, the array becomes softer with a *k* slowly decreasing to 0.008 pN/nm (see Figure 3A). Again this value agrees with the progressive change of slope in the force extension curve that can been seen when the total extension reaches 150 nm ((2) on Figure 2B). For *α* further decreasing from 0^*o*^to -90^*o*^, the progressive rupture of contacts at SHL 5.5 and -5.5 combined with the softening of the array results in the ‘force plateau’ in Figure 3B of Kruithof’s paper ((3) on Figure 2B). In this frame, the energy of interaction they attribute to nucleosome stacking can be attributed to the DNA/histone contacts at SHL 5.5 and -5.5. For a more quantitative estimation of this energy, the reader can refer to the next section.

Our calculations also confirm the three fold increase in stiffness measured for a NRL of 167 bp (see Figure 3B), which is essentially due to the rapid increase of the stretch modulus with the linker length reduction. The measured stiffness of the array (*k*=0.05pN/nm) is compatible with an *α*angle of about 30^*o*^, suggesting that the fiber with shorter NRL has a more open conformation of the entry/exit linkers. Taken together, all these calculations strongly suggest that the mechanical properties of the fiber result from the mechanical properties of the DNA linkers only and not from nucleosome unstacking.

### Extracting the unwrapping energy using our tunable spring model

In the previous sections, we showed that the spring-like behaviour of chromatin fibers under tension can be explained by the mechanical properties of the zig-zag structure of the nucleosomal array combined with the nucleosomal DNA unwrapping. We wish now to give more quantitative details about the elongation of the fiber without linker histones in the presence of magnesium. Our aim is to determine the energies involved in this process. In order to do this, we propose a physical model, similar to the model proposed by Kruithof *et al.*[3]. In this model, we assume two states for the nucleosomes: a crossed or wrapped state, in which *α* is higher than 20^*o*^ (state 1) and an unwrapped state in which *α* is lower than 20^*o*^ (state 2). For each value of the force *f *, there will be *n*_1_nucleosomes in state 1 and *n*_2_=*N*−*n*_1_nucleosomes in state 2 where *N*=25 is the total number of nucleosomes in the array. The elongation *z* can then be calculated as:

(1) $$z(f)=n_{1}(f)d_{1}(f)+n_{2}(f)d_{2}(f)$$

where *d*_1_(*f*) and *d*_2_(*f*) are the length of the fiber per nucleosome respectively in states 1 and 2. Those lengths can be estimated knowing the spring constants *k*_1_and *k*_2_ of the fiber respectively in states 1 and 2.

(2) $$d_{i}\left(f\right)=d_{i}\left(0\right)+\frac{f}{k_{i}},\;\;i=1,2.$$

On the other hand, *n*_1_(*f*) and *n*_2_(*f*) can be calculated from the free energies *F*_1_(0) and *F*_2_(0) of the two states for *f*=0 according to their Boltzmann factors:

(3) $$n_{i}\left(f\right)=N\frac{e^{-G_{i}/k_{\mathrm{BT}}}}{e^{-G_{1}/k_{\mathrm{BT}}}+e^{-G_{2}/k_{\mathrm{BT}}}},\;\;i=1,2$$

where

(4) $$\begin{array}{l} G_{i}(f)=F_{i}(f)-fd_{i}(f)\\ \;=F_{i}(0)+\frac{1}{2}k_{i}{\left(d_{i}(f)-d_{i}(0)\right)}^{2}-fd_{i}(f),\;\;i=1,2 \end{array}$$

are the free enthalpies of the two states. *E*_0_=*F*_2_(0)−*F*_1_(0) is the energy difference between the two states, or unwrapping energy.

**Figure 4:**
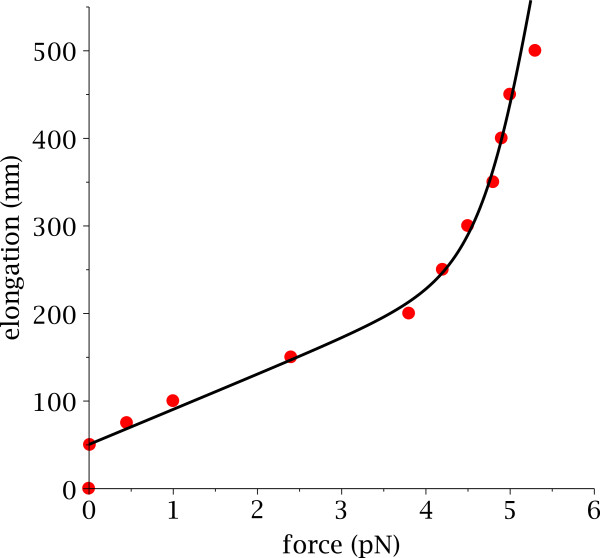
**Fitting our two springs model to the experimental data.** In red the experimental force/extension curve obtained for the 197 bp NRL nucleosomal array, without linker histones and with magnesium. The black line corresponds to the best fit which can be obtained using our two states model.

Fitting the parameters *k*_1_, *k*_2_, *d*_1_, *d*_2_, and *E*_0_to the experimental data (see Figure 4) we found that the two corresponding states were *α*=60^*o*^ for state 1 and *α*=0^*o*^ for state 2. This correspond to an extension of 8 nm per nucleosmes between the two states. Finally, the energy difference between those states was found to be 8*kT*. The exact structural nature of this transition remains to be determined, but it is tempting to hypothesize that this energy corresponds to the disruption of the 6.5 and 5.5 SHL and the disruption of the DNA linker crossing which can be stabilized by the presence of Mg^2+^.

## Conclusion

In conclusion, all the data presented in the Kruithof *et al.* paper [3] can be quantitatively explained by the zig-zag model of fiber morphology. The very high resolution of the experimental data they achieved using their ingenious set up can be used together with the model proposed here to determine very accurately the physical properties of the DNA/histones interactions in chromatin.

## Methods

### The two angle model

To construct our 3D models of the chromatin fiber we used the two–angle model as defined on Figure 1. The 3D structures were created using Maple (http://www.maplesoft.com/). In the present analysis we only considered regular fibers.

### Calculation of the fiber’s stiffness

All the details of the calculations used here can be found in [15]. The relevant elastic constant here is the *effective stretch modulus*, since the nicked DNA in the construct is free to rotate. The stiffness, as measured by Kruithof and colleagues, is equal to the effective stretch modulus divided by the array length. The stiffness depends on both *α* and *β*. Since the *β* angle can change slightly due to the amount of supercoiling stored into the nucleosome, we were able only to provide a maximum and a minimum value for *k* for any given *α*(allowed values for *k* are represented by the grey area on Figure 3).

## Competing interests

The authors declare that they have no competing interests.

## Authors’ contributions

JMV, MB and JM did the analysis. JMV, MB, JZ and JM wrote the manuscript. All authors read and approved the final manuscript.
